# Treatment of agitation in terminally ill patients with intranasal midazolam versus subcutaneous midazolam: study protocol for a randomised controlled open-label monocentric trial (MinTU Study)

**DOI:** 10.1186/s12904-023-01330-1

**Published:** 2024-01-03

**Authors:** Hanna Hirschinger, Evelyn Jaeger, Stefanie Nittka, Svetlana Hetjens, Christine Lorenz, Constanze Remi, Susanne Saussele, Wolf-K. Hofmann, Deniz Gencer, Tobias Boch

**Affiliations:** 1 3rd Department of Medicine, Medical Faculty Mannheim, University Medical Center Mannheim, Heidelberg University, Theodor-Kutzer-Ufer 1-3, 68167 Mannheim, Germany; 2 Institute for Clinical Chemistry, Medical Faculty Mannheim, University Medical Center Mannheim, Heidelberg University, Theodor-Kutzer-Ufer 1-3, 68167 Mannheim, Germany; 3 Department of Medical Statistics, Biomathematics and Information Processing, University Medical Center, Heidelberg University, Mannheim, Heidelberg Germany; 4 Pharmacy, Mannheim University Hospital, Theodor-Kutzer- Ufer 1-3, 68167 Mannheim, Germany; 5 Hospital Pharmacy, Department of Palliative Medicine, LMU University Hospital, LMU Munich, Munich, Germany; 6 Centre for Integrative Oncology, Pain and Palliative Medicine, Oeschelbronn Clinic, 75223 Niefern-Oeschelbronn, Germany

**Keywords:** Palliative care, Terminal agitation, Midazolam, Intranasal, Nasal spray

## Abstract

**Background:**

Intranasal (i.n.) drug application is a widely known and low-invasive route of administration that may be able to achieve rapid symptom control in terminally ill patients. According to the German S3 guideline “Palliative care for patients with incurable cancer”, benzodiazepines, such as midazolam, are recommended for the treatment of terminal agitation. To the best of our knowledge there is no evidence for i.n. midazolam in terminally ill patients. We aim to assess the use of i.n. midazolam as an alternative to subcutaneous administration of the drug.

**Methods:**

In this monocentric, randomised, controlled, open-label investigator initiated trial, n = 60 patients treated at the palliative care unit of a University Hospital will be treated with 5 mg midazolam i.n. versus 5 mg subcutaneous (s.c.) midazolam in the control arm when terminal agitation occurs (randomly assigned 1:1). The estimated recruitment period is 18 months. Treatment efficacy is defined as an improvement on the Richmond Agitation Sedation Scale (Palliative Version) (RASS-PAL) and a study specific numeric rating scale (NRS) before and after drug administration. Furthermore, plasma concentration determinations of midazolam will be conducted at t_1_ = 0 min, t_2_ = 5 min, and t_3_ = 20 min using liquid chromatography/mass spectrometry (LC-MS). The primary objective is to demonstrate non-inferiority of midazolam i.n. in comparison to midazolam s.c. for the treatment of agitation in terminally ill patients.

**Discussion:**

Midazolam i.n. is expected to achieve at least equivalent reduction of terminal agitation compared to s.c. administration. In addition, plasma concentrations of midazolam i.n. are not expected to be lower than those of midazolam s.c. and the dynamics of the plasma concentration with an earlier increase could be beneficial.

**Trial registration:**

German Clinical Trials Registry DRKS00026775, registered 07.07.2022, Eudra CT No.: 2021-004789-36.

## Background

Approximately 43% of all palliative patients are affected by terminal agitation [1], which can manifest as restlessness, sweating and patients’ statements as verbal or facial expressions and defensive reactions [2]. Symptom-focused treatment aims to reduce the burden on patients and their relatives and to prevent the associated reciprocal transmission of anxiety and agitation [3].

First, potential causes of agitation, such as inadequately controlled pain, obstipation, or urinary retention need to be identified and treated. Non-pharmacological measures, such as creating a calm environment, trust-building communication and continuity of care are essential. For the pharmacological treatment of terminal agitation, the German S3 guideline “Palliative Care for Patients with incurable Cancer” recommends the use of midazolam as s.c. or intravenous (i.v.) injection at a dose of 1–5 mg/single dose [2].

Particularly at the end of life, palliative care options are challenged by the patient’s declining organ function, limited time for treatment attempts, and reduced options for drug administration, e.g. due to dysphagia [4, 5]. As the vast majority of home caring relatives do not have a professional medical background, simple and less invasive routes of drug administration are preferred in the home care setting.

Studies have shown that the i.n. drug administration is a convenient and safe alternative route of administration in palliative care [6, 7]. A ready-to-use nasal spray does not require any preparation efforts prior to administration, unlike the injection solutions available for s.c. or i.v. drug administration. Administration is less time-consuming, less complicated and requires little or no prior medical knowledge [6, 8]. At the same time, the i.n. administration is often associated with a rapid therapeutic effect due to the direct nasal absorption of the drug into the bloodstream, similar to the pharmacokinetics after i.v. administration [9]. In previous publications, midazolam i.n. was well tolerated. In these studies investigating the efficacy and tolerability of midazolam i.n. in other patient groups and other indications, mild and local transient adverse events such as nasal irritation, watery eyes, bitter taste or dysgeusia and throat irritation have been described [11–19]. These side effects may be explained by the non-physiological pH value of the solution, which may irritate the nasal mucosa [10].

Palliative care patients are a very vulnerable population and there are only few data on pharmacological treatment strategies at the end-of-life. Due to the specific characteristics of this patient group, data from clinical trials with other patient groups can be transferred only with restrictions. Patients at the end of life are often cachectic, the integrity of the nasal mucosa may be impaired and organ functions are limited. Therefore, clinical trials with terminally ill patients are urgently needed to base therapeutic decisions on robust scientific data [11]. Our clinical trial aims to provide evidence of non-inferiority of midazolam i.n. compared to midazolam s.c. in the treatment of agitation in terminally ill patients.

## Methods/Design

### Study design

The MinTU Study is a randomised controlled, monocenter trial conducted at the University Hospital of Mannheim, Germany. Its purpose is to measure the efficacy and tolerability of midazolam i.n. and demonstrate non-inferiority to midazolam s.c. for the treatment of agitation in terminally ill patients. The study has been designed in accordance with the specifications of the German Drug Law [12] and Good Clinical Practice (GCP) guidelines [13]. It has been registered in German Clinical Trials Register with the identification number 00026775 [14], the Eudra CT number is 2021-004789-36.

### Investigational medicinal product

Midazolam nasal spray 25 mg/ml, solution for intranasal use is used as investigational medicinal product (IMP). The midazolam nasal spray formulation from the NRF formulation finder is used [15]: In addition to the active ingredient midazolam, the aqueous solution contains benzalkonium chloride and sodium edetate for preservation and sodium chloride for isotonisation. Because of its pH-dependent solubility, hydrochloric acid is added to the solution to give a slightly acidic pH of about 3.3. It is manufactured by the pharmacy of the University Hospital Erlangen. One puff of the nasal spray, corresponding to a dispensing volume of 0.05 ml of the active ingredient solution, corresponds to a dose of 1.25 mg of midazolam. Four puffs, equivalent to 5 mg of midazolam, should be administered per single dose in the clinical trial.

### Study population

All patients admitted to the palliative care unit at the study site will be offered the opportunity to participate in the study, as patients must be able to give consent. Inclusion and exclusion criteria are described in Table 1. Only patients with terminal agitation, as assessed by the RASS-PAL score, will be treated and therefore randomized to receive midazolam either i.n. or s.c.

**Table 1:** Overview of the inclusion and exclusion criteria of the MinTU study

Inclusion criteria	Exclusion criteria
• Patients receiving specialized palliative care on the palliative care ward at study site	• Impaired nasal absorption or no possibility for subcutaneous application
• Patients aged 18 years or older	• Patients with hypersensitivity to midazolam
• Capacity to give consent: Participants must be able to understand the nature and scope of the clinical trial.	• Patients who have taken midazolam within the past 24 h
• Dying Patients in the terminal phase of their illness	• Intake of strong CYP 3A4 inducers/inhibitors
• Patients experiencing terminal agitation	• Other causes of agitation
• RASS PAL Score greater than 0	• Sufficient effectiveness of non-drug interventions

### Study procedure

All members of the study group (physicians, nurses and pharmacists) have been trained by the investigator in study procedures according to GCP guidelines. All members of the study group meet the standard operating procedures requirements for an appropriate study site.

On admission to the palliative care unit, the physician informs the patient about the clinical trial and offers to participate. After written consent is obtained, the screening phase is conducted: demographic data are collected and the inclusion and exclusion criteria are reviewed.

If treatment relevant agitation occurs during the terminal phase, a second screening is performed, and the inclusion and exclusion criteria are reassessed. The patient gets randomised by the data management office. There is no blinding. In the treatment group, the patient receives 5 mg midazolam as nasal spray, while in the control group, the patient receives 5 mg midazolam as s.c. injection. In both groups the drug is given as a single dose, therefore, there is no need to adjust the dose. The investigator is the person responsible for the administration of the IMP. Therefore, good compliance with the correct study procedure can be assumed.

The RASS-PAL score and the NRS classifications will be assessed three times: Prior to drug administration and at 5 and 20 min after drug administration. Concurrent blood samples are taken at the same three times points, using lithium-heparinzed blood collection tubes with separation gel (Sarstedt-Monovette®), to determine the drug concentration in patient’s blood. This allows us to objectify the efficacy of the investigational medicinal product. For ethical reasons, blood samples are only taken from patients with a permanent venous catheter.

**Fig. 1:**
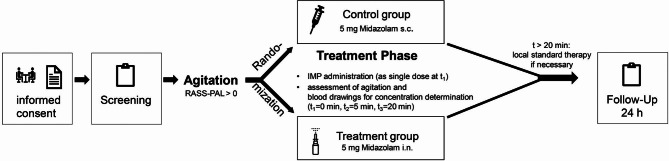
Overview of the study procedures

The treatment phase lasts only 20 min, after which any persistent or recurrent agitation can be treated with standard therapy, midazolam s.c., if necessary. Further follow-up is performed within 24 h of treatment to assess the efficacy and safety of the drug administration. Figure 1 provides an overview of the study procedures.

The investigator may discontinue a patient’s participation in the trial after considering the risk-benefit balance, if therapeutic intervention as defined in the protocol is no longer justified. As patients receive end-of-life treatment, disease progression is not a reason for stopping the trial. Withdrawal from the trial is possible if, after randomisation, the inclusion and/or exclusion criteria are not met. If a study specific treatment is not initiated after a successful initial screening because there is no need for treatment of terminal agitation, this is considered a screening failure.

### Integration of study procedure into regular care

The trial was designed in direct consultation with the medical and nursing staff of a palliative care unit to ensure that the study visits could be seamlessly integrated into routine palliative care.

### Informed consent

All patients admitted to the palliative care unit at the study site will be offered the opportunity to participate in the study and will receive a consent form from an investigator. Patients are given sufficient time to consider their decision and are encouraged to discuss any study-related questions with their physician. If patients agree to participate, they sign the consent form. Patients can withdraw their participation at any time for any reason and without incurring any disadvantage.

### Intervention

Treatment in the clinical trial consists of a single dose of midazolam. Patients will receive either 5 mg midazolam by nasal spray or by s.c. injection. Any persistent or new symptoms after 20 min of administration will be treated according to the standard of care in the palliative care unit.

### Randomisation

Randomisation will take place immediately after or in conjunction with screening part two, if the patient has symptoms of agitation requiring treatment. Randomisation was computerised by an independent statistician from the Department of Medical Statistics, Biomathematics and Information Processing at the University Hospital Mannheim using SAS 9.4 according to the RANUNI statement. Allocation concealment is performed by the data management office, which informs the investigator immediately before the study treatment.

### Outcome measure

Primary outcome: The primary outcome is defined as an improvement of at least one point in the RASS-PAL-Score after drug administration, which is considered as an indicator of drug efficacy.

Secondary outcomes: The secondary outcomes are the detection of a midazolam drug concentration in the blood of c ≥ 30 ng/ml at 20 min, and a quantitative analysis of blood samples at 5 min after drug administration to confirm that the drug has reached the bloodstream.

### Measurement instruments

The RASS-PAL Score [16] and the NRS are used to assess the severity of agitation symptoms. The RASS-PAL Score has been developed to measure the level of agitation. A RASS-PAL score greater than 0 is considered an indication for treatment.

To ensure a consistent assessment of agitation symptoms, a German NRS was developed, validated, and pre-specified in collaboration with the multi-professional team of the palliative care team at the study site (Table 2).

**Table 2:** Trial specific German numeric rating scale to assess the severity of agitation and to evaluate the efficacy of the investigational medicinal product

Restlessness	**3 Severe restlessness** Patient is restless, shows tendency to get up and run away (flight from bed). Is at risk of falling. Patient is agitated, +/- in constant movement. Eyes usually wide open, no eye contact possible and patient strongly disoriented, often calls for the nurse. Patient is very anxious. **2 Restlessness** Increased urge to move, +/- tendency to get up, +/- makes verbal expressions such as “go home”/“(leave here)”. Various signs of anxiety: patient expresses anxiety, +/- anxious look. Patient is partially disoriented. Respiratory rate may be increased. **1 Mild restlessness** Patient calls or rings and does not know why. Still responds or responds again when spoken to. May be tense (facial expression and posture), +/- fidgeting and restless hands. **0 Calm** Patient is calm and relaxed: gestures and facial expressions are relaxed. Patient returns the gaze or is asleep.
Defensive reactions	**3 Strong defence** Patient shows defensive behaviour with hands, +/- feet. Pulling on supply/infusion lines. Attempts to climb over the bed rail. Strong clinging, uncontrolled grasping and grabbing, +/- grabs nurse’s arms. rejects everything and doesn’t tolerate anything in close proximity: Wants everything away from the body and will allow nothing. **2 Defensive** tensing of extremities. Patient becomes “stiff” and cramped, +/- clings to bed rail. **1 Mild defence** Patient turns away. Slight tension when touched or positioned. **0 No defence**
Disorientation	**3 Severe disorientation** Patient is temporally, spatially, +/- personally disoriented. May have delusions and/or hallucinations, +/- feels threatened. May be aggressive. Verbal communication is not possible, patient is no longer communicatively accessible. **2 Disorientation** Patient disoriented in time +/- location. Often rings bell and does not know why. Searches for things and moves around a lot. Hangs on thoughts - “must go to the toilet”/“must call wife”/“must call doctor”. **1 Mild disorientation** Patient notices own disorientation, can be “taught”/disorientation reversible by addressing and explaining, contact is still possible or possible again. **0 Orientation**
Verbal expressions	Patient is restless - “go out”/“go where”/“go home”. Expressions such as: “want to go to a person”/calling out names. Patient feels they need to do something.
Facial expressions	High reddened head, tense jaw muscles, grimaces, forehead wrinkles

The clinical chemistry laboratory at the study site will analyse the blood samples. The samples are centrifuged and stored at -20 °C until they are analysed. After protein precipitation and chromatographic separation, midazolam is analysed by mass spectrometric detection of the ionised analyte mass [M + H] + and quantification using the area under the curve (AUC) of the specific fragment ion (quantifier). The measurement is performed by multiple reaction monitoring with detection of the m/z mass transitions 326 > 291 for midazolam. In this study, the minimum therapeutic concentration of midazolam for the treatment of terminal agitation is set at c ≥ 30 ng/ml [17, 18].

### Data collection

Data collection will begin after informed consent has been obtained. Data collected during the study include patient demographics, treatment and symptom history, observation form data and laboratory parameters (see Table 3). In addition, quantitative analytical evaluation of blood samples is performed to determine drug levels. Non-electronic paper-based CRFs are used for data collection. The data management office collects all data. Access to the data is subject to informed consent. Detailed information on the handling of personal data is provided with the informed consent form.

**Table 3:** Demographic data

Age
Gender
Weight and height
Nutritional status
Laboratory tests: liver and kidney
Distressing symptoms and underlying disease
Concomitant medications
Vital signs: pulse, respiration

Regular monitoring of the trial is performed by an on-site monitor according to the monitoring plan. Adverse events or other unintended effects will be reported to a local medical reviewer. The entire trial from planning to evaluation is overseen by a steering committee formed by interdisciplinary experts.

### Sample size calculation

Due to the lack of research data, it is not possible to estimate the exact data distribution of the study outcomes, such as the extent to which the severity of terminal agitation/restlessness changes after midazolam administration [19, 20]. Therefore, the clinical trial is being conducted as a pilot study. To estimate the sample size of the population, the central limit theorem will be followed. It states that if the sample size n is sufficiently large (approximately n ≥ 25), these mean values will be normally distributed (even if the population is not normally distributed) [21]. A total of 60 patients will be enrolled in the study, with a drop-out rate of 20%. The trial will have two treatment arms, each comprising 30 patients. Assuming that one patient will be treated per week, the recruitment phase will last 18 months.

### Statistical analysis

An independent statistician performs all statistical calculations using SAS 9.4. The analysis will follow the intention-to-treat principle, and all test results received will be included in the analysis. Missing data are reported as such, and if possible, the cause is noted. Quantitative data for approximately normally distributed parameters are presented as means and standard deviations, and medians and ranges for non-normally distributed data. Qualitative data are presented as absolute and relative frequencies. Paired t-tests are used to compare the means of two paired subgroups when the differences are normally distributed, while the Wilcoxon test is used when the differences are not normally distributed. A p-value < 0.05 is considered statistically significant.

### Ethical approval

This trial was approved by the Medical Ethics Commission II of the Medical Faculty Mannheim/University of Heidelberg. Identification number: 2022-4 monocentre.

## Discussion

The MinTU study is an open-label, randomised, controlled trial investigating the effect of midazolam i.n. on terminal agitation in palliative care patients receiving specialized palliative care on the palliative care ward at study site. This manuscript describes our trial, which is investigator-initiated and not commercially sponsored. The trial was conducted solely to advance medical research in the area described and to benefit patients by improving therapeutic options and evidence.

Complex palliative care is tailored to the individual needs of each patient. It often does not follow rigid rules, such as a standardised trial protocol. Therefore, the trial was planned in direct consultation with palliative care physicans and nurses to ensure that the implementation of the study visits could be well integrated into routine palliative care and is feasible.

Patients in the control arm will receive midazolam as s.c. injection. Subcutaneous administration was chosen as the control group based on the fact that the use of a s.c. bolus for rapid symptom reduction has been shown to be effective and can be administered by qualified nursing staff. Compared to i.v. drug administration, there is a lower risk of infection. As midazolam can be given as a parallel s.c. bolus continuous i.v. infusions required for other therapeutic purposes do not need to be interrupted. Midazolam is a well-known agent with sedative and anxiolytic effects. Midazolam is considered the gold standard for the treatment of terminal agitation and anxiety according to the recommendations of the German S3 guideline, although the sedative effect may limit interactions between the patient and their relatives or medical staff at the end of life [2]. The use of midazolam is justified by its short-acting nature and ease of control, with drug therapy most commonly given as a single dose on demand to avoid permanent sedation [18]. The indication for the use of midazolam can be assessed on a patient-by-patient basis using the RASS-PAL score.

While any effective drug therapy carries potential risks of adverse drug reactions, the planned clinical trial is expected to have a calculable risk profile due to the use of an already known drug. The aim of the trial is to investigate the use of midazolam as a short-term on-demand medication for symptom control at the end of life in critically ill patients. The most common side effects expected after administration of the treatment are local. In other studies in other patients and indications, local side effects of midazolam nasal spray have been reported to be acceptable and transient.

In order to minimise the potential additional burden on patients and their families, the study is designed so that the patient is exposed to only minor additional study specific measures. The total number of blood samples taken from the patient during the course of the study is limited to three time points, and blood is only taken from patients with a permanent venous catheter to avoid additional needle sticks.

Patients need to be informed at the earliest possible time point so that they understand the nature and scope of the clinical trial and are able to agree to participate independently. As only patients who are able to give informed consent can be enrolled in the trial, recruiting difficulties are expected during the recruitment phase. Patients may arrive at the site in a reduced state of consciousness, or their general condition may get worse rapidly as the dying process progresses. It may be necessary to extend the recruitment period. The required number of cases is difficult to quantify, as there are few data and no established measurement tools to aid symptom assessment and evaluation of treatment effectiveness for agitation in palliative care. In this trial, the RASS-PAL-Score and an additional NRS will be used. The NRS was developed in collaboration with the palliative care staff using the Delphi method to provide a consistent measurement of symptom severity and to assess the efficacy of the investigational medicinal product.

This trial has certain limitations. It is not placebo-controlled, because patients at the end-of-life should not be deprived of an effective therapy to reduce distressing symptoms. The trial is not blinded because of the need for a double dummy, which could be an additional burden on the patient and should be avoided.

There is currently no data available for the patient population and indication studied in our trial. Therefore, treatment decisions are based on extrapolated data from other trials in different patients and indications, as well as the experience of clinicians. However, scientific data from clinical trials are necessary to practice evidence-based medicine and provide appropriate support to terminally ill patients. Such data are essential to ensure that patients receive the best possible care based on reliable evidence.

## Conclusion

It is expected that midazolam i.n. will achieve at least an equivalent reduction in terminal agitation as midazolam s.c. Furthermore, plasma concentrations of midazolam i.n. are not expected to be lower than those of midazolam s.c.

The intranasal route of administration could serve as a potential alternative to the subcutaneous route, particularly to simplify palliative care at home. If proven effective, this would greatly facilitate palliative care by offering a quick and easy symptom control that requires little or no prior medical knowledge or training of the user.

The MinTU study began recruitment in August 2022 and is expected to be completed within 18 months.

## Data Availability

Data sharing is not yet applicable to this article as no datasets have been generated or analysed at this point of the current study. The datasets used and/or analysed during the current study are available from the corresponding author upon reasonable request.

## List of abbreviations

AUC: Area under the curve
CRF: Case report form
GCP: Good Clinical Practice
i.n.: Intranasal
IIT: Investigator initiated trial
IMP: Investigational Medicinal Product
LC-MS: Liquid chromatography-mass spectrometry
NRS: Numeric rating scale
RASS-PAL: Richmond Agitation Sedation Scale - Palliative Version
s.c.: Subcutaneous
